# Squamous cell carcinoma of the hypopharynx in a young woman with Fanconi’s anemia

**DOI:** 10.1016/S1808-8694(15)31054-5

**Published:** 2015-10-19

**Authors:** Henrique de Lins e Horta, Fernando Fernandes Guimarães, Luiz Otávio Savassi Rocha, Roberto Eustáquio Santos Guimarães, Eugênia Ribeiro Valadares

**Affiliations:** 1 MD. General Practice Resident - University Hospital - Federal University of Minas Gerais.; 2 MD. Otorhinolaryngology Resident - University Hospital - Federal University of Minas Gerais - UFMG.; 3 Associate Professor - Department of Clinical Medicine - Medical School - Federal University of Minas Gerais - UFMG.; 4 Associate Professor - Department of Otorhinolaryngology - Federal University of Minas Gerais - UFMG.; 5 Associate Professor - Department of Pediatrics - Medical School of the Federal University of Minas Gerais - UFMG / PhD in Genetics.

**Keywords:** fanconi’s anemia, squamous cell carcinoma, hypopharynx

## Abstract

Fanconi’s anemia is a rare autosomal recessive disorder characterized by congenital malformation, bone marrow failure and genomic instability, with a predisposition to develop malignancies, especially the leukemias and upper aerodigestive tract tumors. Due to inherent characteristics to this syndrome, the treatment of such neoplasms is particularly difficult. In this paper we report the case of a 24-year-old woman with Fanconi’s anemia who developed a squamous cell carcinoma of the hypopharynx; she had none of the traditional risk factors, such as smoking and alcohol abuse. We also briefly review the literature about this topic

## INTRODUCTION

Fanconi’s anemia (McKusick 227650), also known as hereditary pancytopenia, is a syndrome characterized by blood disorders associated to congenital malformations, of recessive autosomal inheritance, with clinically variable expression. Among the most characteristic clinical manifestations we have growth retardation, hypoplastic thumbs (or absent) and short radius (or absent). Alongside other hereditary disorders such as Bloom’s syndrome and ataxia telangiectasia, Fanconi’s anemia is part of the group of syndromes marked by genomic instability, and this predisposes patients to different types of cancer. In the cytogenetic study, the genomic instability is manifested as breaks and rearrangements in different chromosomes.

Fanconi’s anemia treatment is based on support measures, medication (steroids, androgens, colony stimulating factors) and, in selected cases, bone marrow allogenic transplant. Most patients die due to pancytopenia-related blood complications or sepsis. On the other hand, those who develop malignant neoplasias, and are not proper candidates to surgical treatment, are more prone to the adverse effects of radiotherapy and chemotherapy, in such a way as to make it even more difficult to plan for therapy.

Hereto, we make a summary review of the literature and report a case of squamous cell carcinoma of the hypopharynx in a young adult with Fanconi’s anemia.

## LITERATURE REVIEW

First described in 1927, Fanconi’s anemia is a rare recessive autosomal disorder, with heterogenous genotype and phenotype, characterized by constitutional aplastic anemia, congenital malformations, chromosome instability and predisposition to the development of neoplasia.^1^, ^2^ Clinical manifestations vary, and include: growth and sexual maturation retardation, low stature, skeletal abnormalities (radius and thumb), bone marrow aplasia, café au lait spots and renal and cardiac malformations. Diagnosis is carried out by a cytogenetic analysis that shows a greater trend towards chromosome rupture by alkylating agents such as mitomycin C and diepoxybutane.

The genetic basis for Fanconi’s anemia is as complex as its great variation in clinical presentation. There are at least eight subtypes (A, B, C, D1, D2, E, F and G), each one with a distinct genetic defect. Pathogenesis is related to the lack of DNA repair mechanisms, with the consequent build up of a large number of mutations. Recent studies suggest that Fanconi’s anemia genes, such as the genes BRCA1 and BRCA2, act on a final common pathway, related to DNA repair.^3^

Because of this incapacity in keeping genomic integrity, we see a high degree of chromosome instability. Patients have a higher risk of developing neoplasia, which increases with the increase in life expectancy.^4^ The most prevalent type is leukemia (especially acute myeloid); followed in a descending order by squamous cells carcinoma (aerodigestive tract, anus, genital area and skin), and brain, liver and renal neoplasias.^5^ Neoplasias usually are seen when the patient is about 16 years of age, and with age increase it is not uncommon to see other tumor types affecting the same person.6 Moreover, there are case reports in which the cancer diagnosis preceded that of the syndrome.

Fanconi’s anemia-associated solid tumors treatment is primarily surgical, even with a higher operative risk because of bone marrow aplasia.^7^ Epidermoid carcinomas of the head and neck are usually more biologically aggressive, in such a way as to require adjuvant therapy. However, because of defects on the DNA repair mechanisms, patients are very susceptible to radio and chemotherapy side effects, especially myelosuppression. Even so, these therapy modalities must be considered when surgery is contra-indicated. In such cases, we recommend using doses lower than the ones commonly used, tailor-made regimens (that do not include alkylating agents or cisplatin) and the use of radio-protective agents such as amifostine.^8^, ^9^, ^10^

## CASE REPORT

A 24 year old brown and single woman, a student that after being diagnosed with Fanconi’s anemia when she was 12 years old, started to make regular use of medroxyprogesterone and oxymetholone. There are two other cases diagnosed in her family: a 21 year old sister, and a brother who died at 15 years of age victim of leukemia. Her parents are related by kinship.

**Figure 1:**
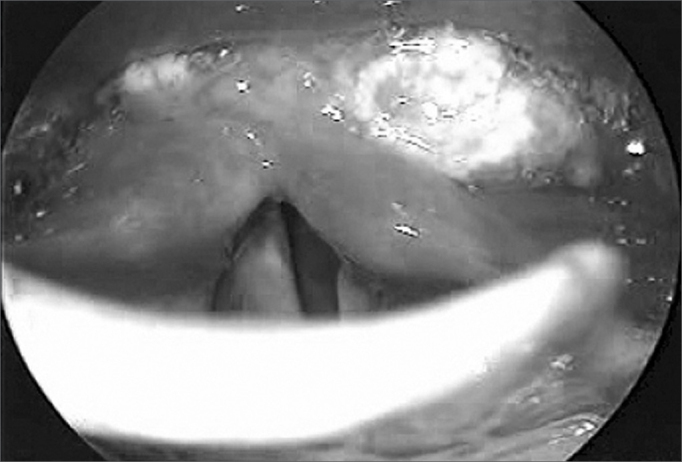
Tumor view at videolaryngoscopy: vegetating lesion involving mostly the left piriform cavity.

**Figure 2:**
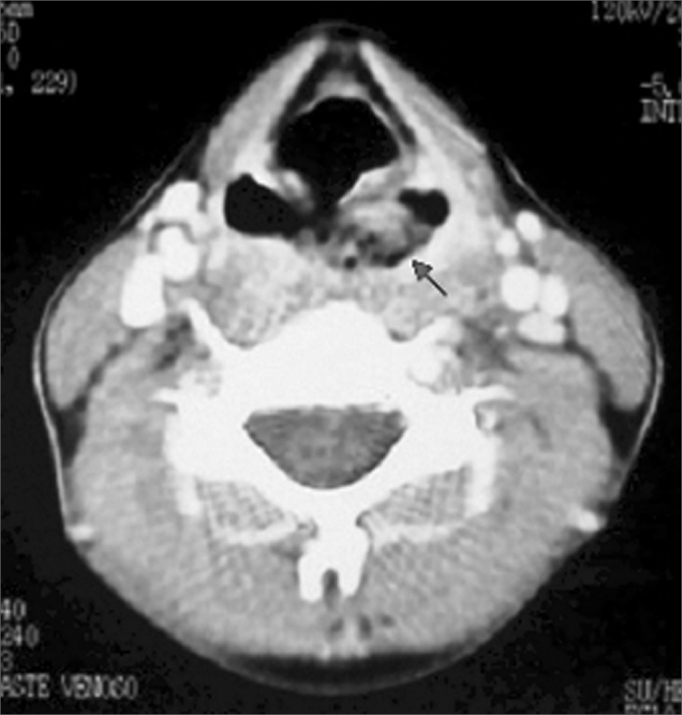
Neck CT scan, axial view: tumor mass in the left piriform cavity.

The patient, who did not drink or smoke, was admitted to a University Hospital because of progressive hoarseness, dysphagia, odynophagia and weight loss, with six months of evolution. Initial clinical exam showed low weight and height development (weight = 28.7 kg) (height= 1.32 m), café au lait spots in her torso and malnutrition. CBC showed important pancytopenia: hemoglobin= 5.9 g/dl, leukocyte count= 800/mm^3^ and platelets = 29.100/mm^3^. Videolaryngoscopy revealed a vegetant and ulcerated lesion, mostly located on the left piriform cavity, invading the proximal esophagus (Figure 1). A neck CT scan showed a soft mass in the hypopharynx area involving the left piriform cavity and going from the aryepiglottic folds all the way to the arytenoid cartilages, with a probable segmentary concentric parietal thickening of the upper esophagus. We did not observe enlarged lymphnodes (Figure 2). Lesion biopsy revealed an invasive, ulcerated and moderately differentiated squamous cell carcinoma.

During intervention, the patient presented numerous fever episodes with neutropenia, and this caused the prescription of many antibiotic agents in association (ceftazidime, amikacin, imipenem, vancomycin, amphotericin B e fluconazole).

The possibility of surgical treatment (total pharyngoesophagectomy with the placement of a retro-sternal gastric tube, bilateral lymphnode clearance, partial thyroidectomy and postop radiotherapy) was ruled out since the patient did not allow it and would not tolerate it. Oncologists counter-indicated chemotherapy, and only palliative radiotherapy was used.

Coinciding with radiotherapy, there was a worsening in dysphagia and odynophagia, and the fever returned, which led us to discontinue it (total dose of radiotherapy received was 19.8 Gy). The patient developed pneumonia together with severe neutropenia (leucocytes 100/mm^3^), and died despite treatment with antibiotics.

## DISCUSSION

In a large number of cases, squamous cell carcinomas of the hypopharynx are closely related to tobacco and alcohol exposure, usually affecting those above 60 years of age, mainly males.^11^ In the case hereby reported - a young 24 year old patient, without prior exposure to alcohol or tobacco - only the concurrence of another predisposing factor - Fanconi’s anemia, could have explained this type of neoplasia. In a study in which they assessed 754 patients with this syndrome, the authors noticed an increase of approximately 500 fold in the incidence of head and neck tumors, mainly in females (2:1), and average age at diagnosis of 31 years, when compared to the general population.^12^

## FINAL COMMENTS

Having seen the great variation in clinical presentation, the few cases reported in the literature and the high morbidity associated to the different forms of treatment, we appreciate the great difficulty in approaching head and neck neoplasias associated to Fanconi’s anemia. Therefore, all effort should be put up in order to detect it early on by means of a periodic medical evaluation that must include a thorough otorhinolaryngological assessment.
